# Effects of ultrasound-guided serratus plane block combined with general anesthesia on postoperative early quality of recovery and analgesia in patients undergoing transapical transcatheter aortic valve implantation surgery: study protocol for a randomized controlled trial

**DOI:** 10.1186/s13063-024-08252-0

**Published:** 2024-07-02

**Authors:** Cheng Xiao, Fang Chen, Lei Cao, Ming Yang, Yuting Tan, Guoyun Lin, Guiying Yang, Sheng Jing, Hong Li

**Affiliations:** Department of Anesthesiology, Xinqiao Hospital of Chongqing, Second Affiliated Hospital of Army Medical University, PLA, Chongqing, 400037 China

**Keywords:** Serratus anterior plane block, Transcatheter aortic valve implantation, Postoperative recovery quality, Analgesia

## Abstract

**Background:**

Compared to traditional thoracotomy, transapical transcatheter aortic valve implantation (TAVI) surgery offers reduced trauma and faster recovery, fostering the adoption of enhanced recovery after surgery (ERAS) protocols in cardiac surgery. Despite these advancements, postoperative pain management has received insufficient attention. The potential effects of multi-mode analgesia, including ultrasound-guided serratus anterior plane block (SAPB), on postoperative pain and early quality of recovery have not been widely studied, lacking comprehensive prospective evidence. Therefore, this study aims to investigate the impact of SAPB combined with general anesthesia on early recovery quality and analgesic efficacy in transapical TAVI patients.

**Methods:**

This prospective, randomized controlled study will enroll 70 patients undergoing transapical TAVI, randomly allocated to either the SAPB group or the control group. The primary outcome, assessed using Quality of Recovery-40 (QOR-40) scale, focuses on the quality of recovery at 24 h and 48 h postoperatively. Secondary outcomes include the visual analog scale (VAS) pain scores at rest and during coughing at 6 h, 12 h, 24 h, and 48 h after surgery, frequency of patient-controlled analgesia (PCA) utilization at 24 h and 48 h, opioid consumption at 24 h and 48 h, time and frequency of rescue analgesia and severe pain at 24 h and 48 h, incidence of nausea and vomiting at 48 h after surgery, and dosage of antiemetic drugs.

**Discussion:**

The purpose of our study is to evaluate the effects of ultrasound-guided SAPB combined with general anesthesia on postoperative early quality of recovery and analgesia in transapical TAVI patients. The results obtained may provide valuable insight for the implementation of multi-mode analgesia and enhanced ERAS in this specific patient population.

**Trial registration:**

China Clinical Trial Register ChiCTR2300068584. Registered on 24 February 2023.

## Introduction

### Background and rationale {6a}

Aortic valve disease is a prevalent valvular disorder encountered in clinical settings. Traditionally, the mainstay treatment for improving patient outcomes has been open-heart aortic valve replacement surgery, a procedure characterized by its highly invasiveness, requiring cardiopulmonary bypass and cardiac arrest. This approach, particularly challenging for elderly patients or those with multiple comorbidities, was associated with considerable early postoperative mortality rates. The breakthrough came with the first TAVI procedure in France in 2002, revolutionizing heart valve replacement through an interventional approach [1].

In recent years, TAVI has gained popularity, particularly suitable for high-risk patients [2]. Transapical TAVI presents several advantages, including operating in the direction of blood flow, shorter procedural distances, fewer intracardiac manipulations, reduced valve displacement risks, and the ability to overcome poor peripheral vascular conditions. Additionally, it significantly decreases contrast agent usage and exhibits lower incidences of stroke and organ embolisms [3]. Despite its advantages, including minimal incision and fast recovery compared to traditional surgery, postoperative pain remains a significant challenge.

Ultrasound-guided regional blocks, crucial components of multimodal analgesia, are widely utilized in routine clinical practice. SAPB is a chest wall nerve blockade technique [4], wherein local anesthetics (LAs) are diffused into the plane of serratus anterior muscle under ultrasound guidance. This effectively blocks the lateral cutaneous branches of intercostal nerves [5], infiltrating the long thoracic and thoracodorsal nerves, providing analgesia to the anterolateral chest wall. SAPB also assists in reducing the secretion of postoperative pain mediator, suppressing inflammatory responses, and decreasing inflammatory cytokine release. Additionally, it can reduce opioid consumption and its associated adverse effects, which is particularly relevant for elderly patients, especially those with severe systemic complications, thus contributing to the quality of postoperative recovery. Its application spans breast surgeries, thoracic surgeries, and rib fracture surgeries [6, 7]. However, the literature on the application of ultrasound-guided SAPB in minimally invasive cardiac surgeries, especially transapical TAVI, remains limited. Berthoud et al. [8] and Peng et al. [9] reported patients successfully completed apical TAVI surgery under SAPB due to serious complications. Furthermore, Berthoud et al. compared the therapeutic effects of SAPB and local incision infiltration for postoperative pain management in minimally invasive cardiac surgery. The results showed that SAPB can effectively alleviate postoperative pain in patients undergoing minimally invasive cardiac surgery and positively impact patient prognosis [10].

Furthermore, the integration of multimodal analgesia with regional block technique can improve analgesic efficacy while mitigating the adverse effects associated with opioid analgesics. However, achieving meaningful clinical outcomes require optimal postoperative recovery. Anesthesia and postoperative recovery constitute a multidimensional and intricate process. The QOR-40 scale is widely utilized in clinical settings to evaluate early postoperative recovery quality following anesthesia and surgery. Recognized for its suitability, feasibility, effectiveness, reliability, and precision, the QOR-40 scale is considered an optimal standard for assessing postoperative recovery [11]. Additionally, researchers have identified that preoperative low QoR-40 scores can predict poor postoperative recovery, and early effective interventions can enhance postoperative recovery quality, thus improving patient quality of life [12]. Therefore, establishing a direct correlation between ultrasound-guided SAPB and post-transapical TAVI surgery recovery quality necessitates further prospective data.

Our study seeks to examine the impact of ultrasound-guided SAPB on early postoperative recovery quality and analgesic efficacy in patients undergoing transapical TAVI procedures. By refining further multi-mode analgesia and ERAS protocols of this surgery, we aim to improve the postoperative rehabilitation quality and optimize the utilization of medical resources.

### Objectives {7}

The aim of this study is to investigate the influence of SAPB combined with general anesthesia on early recovery quality and analgesic efficacy in patients undergoing transapical TAVI.

### Trial design {8}

**Fig. 1:**
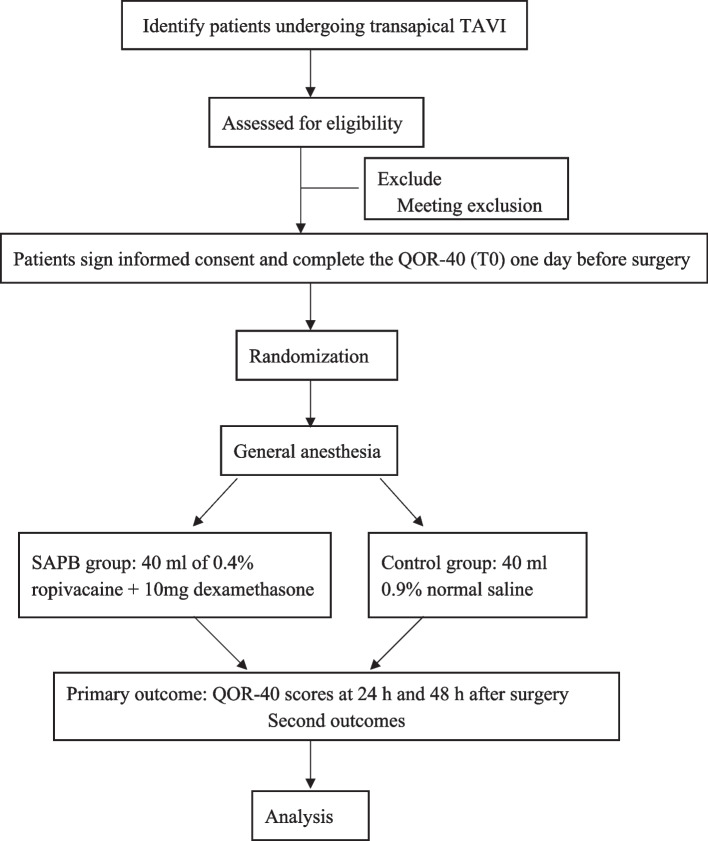
Study flow chart

This is single-center, double-blind, randomized controlled trial aimed at investigating the impact of ultrasound-guided SAPB on early postoperative recovery quality and analgesic effectiveness in patients undergoing transapical TAVI. The study flow chart is depicted in Fig. 1. Our research received funding support from the National Natural Science Foundation of China (Project No. 82171265).

## Methods: participants, interventions, and outcomes

### Study setting {9}

The study will be conducted at the Second Affiliated Hospital of Army Medical University, Chinese People’s Liberation Army (PLA) (tertiary hospital), Chongqing, China. Participant enrollment began in February 2023. The protocol adheres to the principles outlined in the Declaration of Helsinki and follows the Standard Protocol Items: Recommendations for Interventional Trials (SPIRIT).

### Eligibility criteria {10}

#### Inclusion criteria

Patients will undergo a face-to-face screening 1 day prior to surgery based on the following eligibility criteria:

1. Adult patients aged40–70 years
2. ASA II ~ IV classification
3. Patients planning to undergo transapical TAVI under general anesthesia
4. Patients expressing willingness to participate in this study and signed informed consent

#### Exclusion criteria

Patients meeting any of the following criteria will be excluded from the study:

1. Allergy to LAs
2. History of mental illness or taking psychotropic drugs
3. Severe abnormalities in coagulation function
4. Long-term use of analgesics or sedatives
5. History of drug abuse, alcohol, or opioid abuse
6. Recent acute pain or chronic pain
7. Postoperative mechanical ventilation ≥ 24 h
8. Decline to participate in the study
9. Participants retain the right to withdraw from the study at their discretion for any reason or if researchers determine their inclusion is inappropriate

### Who will take informed consent? {26a}

Enrollment of eligible participants will be supervised by a designated member of the research team. Following a comprehensive oral explanation of the study, including its potential benefits and risks, patients or their legally authorized representative will be asked to sign the informed consent form approved by the institutional review committee. Participants and their legal authorized representatives will be informed that participation is entirely voluntary, and they have the right to withdraw from the trial at any time.

### Additional consent provisions for collection and use of participant data and biological specimens {26b}

Not applicable. We will not utilize the data and biological specimens of participants for ancillary studies.

### Interventions

#### Explanation for the choice of comparators {6b}

Currently, there is limited research on the analgesic effectiveness of SAPB in transapical TAVI. Establishing a direct correlation between SAPB and the quality of recovery following transapical TAVI surgery requires more prospective data for validation. This study aims to investigate the impact of SAPB on the postoperative early recovery quality and analgesic efficacy in patients undergoing transapical TAVI surgery, and to solve the contradiction between the patients who cannot tolerate large doses of opioids and the huge hemodynamic changes due to pain and subsequently affecting postoperative recovery quality. The study seeks to optimize the multi-mode analgesia and ERAS for this operation, with the goal of enhancing the postoperative recovery quality and optimizing the medical resources utilization.

Currently, other studies on regional block have used 0.9% saline as control, making it ethically feasible to have a blank control group in this study.

#### Intervention description {11a}

Ultrasound-guided SAPB will be conducted after induction of general anesthesia. Patient will be positioned supine with the upper limb abducted, and the ultrasonic probe will be located in the 5th intercostal space along the left axillary line. The probe’s direction will be adjusted to identify the tissue structures such as latissimus dorsi, pectoralis major, pectoralis minor, and serratus anterior. Using an in-plane technique, a 22-G nerve block needle will be advanced to the surface of the serratus anterior muscle and aspirated to ensure no blood and gas. Subsequently, 2 mL of local anesthetic mixture (0.4% ropivacaine + 10 mg dexamethasone) will be slowly injected. The injection will be performed while observing the spread of the anesthetic between the fascia layers, as indicated by a hypoechoic area on ultrasound, and additional mixture will be slowly injected. The control group will be given 40 mL normal saline.

#### Criteria for discontinuing or modifying allocated interventions {11b}

Withdrawal criteria are as the follows:

1. Patient or legal representative requests to withdraw from the study.
2. Changes in the surgical procedure may occur during surgery.
3. The investigator may decide to terminate the trial due to other unforeseen reasons.

#### Strategies to improve adherence to interventions {11c}

All SAPB procedures, surgeries, and assessments will be consistently performed by the same anesthesiologist, surgeon, and assessor, respectively. Randomization and monitoring processes will be conducted by independent researchers to ensure fairness and protocol adherence. The principal investigator will be responsible for all aspects of the recruitment process and any amendment to the study. All the experiments will strictly adhere to the research plan, ensuring reliability and effectiveness of our results.

#### Relevant concomitant care permitted or prohibited during the trial {11d}

Patients will be prepared in accordance with cardiopulmonary bypass, and standard monitoring will be established, including electrocardiograph (ECG), non-invasive blood pressure (NIBP), pulse oxygen saturation (SpO_2_), body temperature (T), bispectral index (BIS), and cerebral oxygen, with an external automatic defibrillation patch attached. Radial artery and right internal jugular vein catheterization, under local anesthesia, will facilitate monitoring of invasive arteriovenous pressure. General anesthesia induction will involve midazolam (0.03 ~ 0.08 mg/kg), etomidate (0.2 ~ 0.6 mg/kg), rocuronium (0.6 ~ 0.9 mg/kg), and sufentanil (0.3 ~ 0.4 μg/kg). Tracheal tubes will be inserted when the BIS value decreased to 40 ~ 50, initiating mechanical ventilation with a tidal volume of 6 to 8 mL/kg, respiratory rate of 12 to 15/min, and a 1:1 air-oxygen mixture, maintaining ETCO_2_ between 35 and 45 mmHg.

The anesthesia will be maintained with propofol (4 ~ 6 mg/kg/h), remifentanil (0.08 ~ 0.3 µg/kg/min), and sevoflurane (1 ~ 2 vol%). Heart rate and mean arterial pressure will be maintained within ± 20% of baseline, except during rapid ventricular pacing. Blood potassium level will be maintained between 4.5 and 5.5 mmol/L and BIS will be kept between 40 and 60 throughout operation.

Patients will be delivered to cardiac surgery intensive care unit postoperatively for standardized monitoring and management. A postoperative patient-controlled intravenous analgesia (PCIA) regimen will be established as part of a multimodal pain regimen, with sufentanil (2.5 µg/mL, limit dose 200 µg) combined with dexmedetomidine (3 µg/mL, limit dose 200 µg) and tropisetron 5 mg. The PCIA pump will be programmed for a 2 mL bolus with a lockout interval of 15 min and a basal infusion of 4 mL/h. Rescue analgesia will include paracetamol and tramadol hydrochloride tablets (one tablet, p.o) for VAS > 4 at rest, and 1 mg of hydromorphone intravenously for severe pain (VAS > 7). Patients will be educated on PCIA pump usage.

#### Provisions for post-trial care {30}

All study participants will receive standard care. We will closely monitor them throughout the postoperative period to promptly identify any potential complications and implement intervention measures as necessary.

### Outcomes {12}

#### Primary and secondary outcomes

The primary outcome will assess the quality of recovery at 24 h and 48 h, measured using the QoR-40 scale [11, 13].

The secondary outcomes include:

1. VAS pain scores: assessment at rest and during coughing at 6 h, 12 h, 24 h, and 48 h post-surgery [9]
2. Frequency of PCA utilization: evaluation at 24 h and 48 h following surgery [14, 15]
3. Opioid consumption: quantification at 24 h and 48 h post-surgery [14, 15]
4. Time and frequency of rescue analgesia and severe pain: analysis at 24 h and 48 h after surgery [16]
5. Incidence of nausea and vomiting: determination at 48 h post-surgery, along with the dosage of antiemetic drugs [15]

### Participant timeline {13}

The schedule of enrollment, interventions, and assessments is shown in Table 1.

**Table 1:** Participant timeline

	**Enrolment**	**Allocation**	**Post-allocation**			
Timepoint	** − 1 day**	**Surgery day**	**6 h after surgery**	**12 h after surgery**	**24 h after surgery**	**48 h after surgery**
Enrollment						
*Eligibility screen*	X					
*Informed consent*	X					
Allocation		X				
Interventions						
*SAPB group*		X				
*Control group*		X				
Assessments						
*QOR-40*	X				X	X
*VAS pain scores*			X	X	X	X
*Times of PCA*					X	X
*Opioid consumption*					X	X
*Rescue analgesia*					X	X
*Severe pain*						X
*Nausea*						X
*Vomit*						X
*Dosage of antiemetic drugs*						X

*SAPB*, serratus anterior plane block; *QOR-40*, Quality of Recovery-40 scale; *PCA*, patient-controlled analgesia

### Sample size {14}

The sample size calculation is based on the primary outcome. According to a prior publication, where mean of group 1 was 167 and the mean of group 2 was 184, with known group standard deviations of 23 [13], a sample size of 33 for each group (66 in total) will be required to achieve a power of 80% to detect the difference at a two-side *α* level of 0.05, taking into account a dropout rate of 10%. Ultimately, we plan to include 35 participants in each group.

### Recruitment {15}

Patients will be provided with comprehensive information about the research procedures, including potential risks, the benefits, and postoperative follow-up, during pre-anesthesia clinic consultations to ensure thorough understanding and enhance patient compliance. Recruitment will be conducted at the Second Affiliated Hospital of Army Medical University, PLA. The recruitment for this study began on February 28, 2023. Our hospital currently performs 3–4 transapical TAVI procedures per month and has recruited 50 participants to date. To minimize the risk of not reaching the recruitment target, we expect to complete recruitment by January 2025.

### Assignment of interventions: allocation

#### Sequence generation {16a}

Eligible patients will be randomly allocated to either the ultrasound-guided SAPB group or the control group at a 1:1 allocation ratio after providing written informed consent. The randomization sequence will be generated by an independent researcher using SPSS software version 26.0 (IBM, New York, USA).

#### Concealment mechanism {16b}

The randomized results will be securely sealed in opaque envelopes and stored separately until the end of the study. To maintain unbiased assignment and minimize potential confounding factors, the investigators responsible for generating the random sequences will not be involved in recruitment, anesthesia administration, and outcome evaluation. Preoperative interview researchers, unaware of the random allocation number, will screen and recruit participants and completed the QOR-40 scale 1 day before surgery [13].

#### Implementation {16c}

As soon as the patients enters the operation room, the attending anesthesiologist will open the corresponding numbered envelope, verify the patient’s allocation, and proceed to implement the corresponding anesthesia plan accordingly.

### Assignment of interventions: blinding

#### Who will be blinded {17a}

Due to the nature of the study, blinding of the attending anesthesiologist is not feasible. However, patients, outcome assessors, surgeons, and nursing staff will remain blinded until the completion of the study analysis.

#### Procedure for unblinding if needed {17b}

While we do not anticipate the need for unblinding, if necessary, such as the occurrence of a research-related serious adverse event, the principal investigator or data manager will have access to the group assignment and will report any unblinding promptly.

### Data collection and management

#### Plans for assessment and collection of outcomes {18a}

Research data for this study will be collected from electronic medical records systems or case report forms (CRFs). Preoperative data, including demographics, medical history, medication history, supplementary examinations, and preoperative assessment, will be completed by preoperative visitors. The anesthesiologist will record intraoperative data. The QOR-40 scale 1 day before surgery and postoperative outcomes assessment will be collected by a dedicated assessor. To ensure scientific rigor of our study, all researchers will be trained on how to collect, record, and store data before the trial starts. All information will be strictly confidential and used solely for research purpose. After data collection, it will be double-entered into Microsoft Excel system and check by two researchers. The principal investigator will conduct a thorough check for any defects in the raw data again. Participants’ personal information will be kept confidential.

#### Plans to promote participant retention and complete follow-up {18b}

During the preoperative visit, researchers will provide detailed explanation of the intraoperative management and postoperative follow-up process.

#### Data management {19}

All data will be anonymized, with CRFs abbreviated as initials, and electronic data is coded. Electronic data will be stored on a dedicated computer with double password protection, while paper CRFs will be securely stored in our research center with password-locks. All original documents will be retained for 5 years after the study is completed.

#### Confidentiality {27}

Patients will continue to be enrolled until the required number is reached. All data will be anonymized, with CRFs abbreviated as initials, and electronic data is coded. All original documents will only be accessible to researchers with the original ID and password.

#### Plans for collection, laboratory evaluation, and storage of biological specimens for genetic or molecular analysis in this trial/future use {33}

Not applicable. No samples will be collected for this study.

### Statistical methods

#### Statistical methods for primary and secondary outcomes {20a}

All normally distributed continuous variables will be expressed as mean ± standard deviation and analyzed with Student’s *t*-test. For non-normal distributed data, the Mann–Whitney *U* test will be used, and results will be presented as median and interquartile interval. Categorical variables will be described with frequencies (%) and compared using the chi-square test or Fisher’s exact test. A 95% confidence interval (CI) for differences in means (for continuous outcomes) or relative risks (for categorical outcomes) will be calculated. A *P* value < 0.05 will be considered statistically significant. Statistical analyses will be performed using SPSS software version 26 (IBM, New York, USA).

#### Interim analyses {21b}

No interim analyses will be conducted due to the small number of patients and the expected low incidence of serious adverse events.

#### Methods for additional analyses (e.g., subgroup analyses) {20b}

No subgroup analyses are planned.

#### Methods in analysis to handle protocol non-adherence and any statistical methods to handle missing data {20c}

The likelihood of missing values in our primary outcome is very low. If missing data occur, multiple imputation techniques will be employed to accurately estimate them.

#### Plans to give access to the full protocol, participant-level data, and statistical code {31c}

Data sets, statistical codes, and complete protocols analyzed by the institute will be available upon reasonable request to the principal investigator. This approach ensures accuracy, repeatability, and promotes further study and cooperation.

### Oversight and monitoring

#### Composition of the coordinating center and trial steering committee {5d}

The steering committee, composed of principal investigator, sub-investigator, and two clinicians, will guide and supervise the implementation and progress of the trial. Quarterly meetings will be convened to monitor the latest developments in the trial, ensuring that the research design and interpretation remain relevant to current clinical practice. The committee has the authority to make decisions regarding trial modifications based on specific circumstances and assumes medical responsibility for the patients involved.

#### Composition of the data monitoring committee, its role and reporting structure {21a}

This trial is a single-center RCT with relatively small sample size and low intervention risk, and there is no data monitoring committee established. The principal investigator will review all the summarized data, and an independent auditor with expertise will conduct monthly audit. The steering committee will monitor study development quarterly, while the ethics committee will conduct an annual follow-up review.

#### Adverse event reporting and harms {22}

SAPB, integrated into our multi-mode analgesia approach, will be conducted under precise visual ultrasound guidance and standardized skilled operation. Anesthesia safety will be strictly controlled by experienced anesthesiologists within the team. Any adverse effects or unpredictable complications will be diligently documented and promptly reported to the ethics committee as part of our annual report.

#### Frequency and plans for auditing trial conduct {23}

There is no data monitoring committee (DMC) established for this study. Instead, an independent auditor with expertise will conduct monthly audits. Additionally, the steering committee and auditor will perform repeated audits every 3 months to ensure the accuracy and validity of the data. Furthermore, the ethics committee will conduct an annual follow-up review.

#### Plans for communicating important protocol amendments to relevant parties (e.g., trial participants, ethical committees) {25}

Any modifications to the research plan (version 1.0) will be promptly communicated to the principal investigator and the research ethics committee of the second affiliated hospital of army military medical university. A revised plan will be issued following thorough examination and approval.

### Dissemination plans {31a}

The study aims to optimize multi-mode analgesia and enhance ERAS for transapical TAVI, aligning with diagnosis related groups (DRG) in medical management. Results will be disseminated at relevant academic conferences and published in peer-reviewed journals. Both positive and negative findings will be reported.

## Discussion

This single-center, randomized controlled trial aims to investigate the effect of ultrasound-guided SAPB on the early recovery quality and analgesic of patients undergoing transapical TAVI surgery.

Transapical TAVI, a minimally invasive surgical method for aortic valve lesions, offers a promising alternative for elderly and high-risk patients who may not be suitable candidates for traditional surgery. As evidence-based medicine continues to evolve, the indications for TAVI are expanding globally, leading to increased surgical volume and a gradual extension of indications to younger, lower risk, and longer life expectancy groups [17, 18]. However, perioperative analgesia and postoperative recovery of this minimally invasive cardiac surgery have not received widespread attention.

As evidence-based medicine continues to evolve, optimizing postoperative pain management becomes increasingly crucial. A comprehensive and individualized analgesia and rehabilitation program are vital. Perioperative multimodal analgesia, particularly regional block-based approaches, plays a pivotal role in promoting rehabilitation. SAPB is a fascial plane block with minimal impact on peripheral blood vessels and presents a safer and more maneuverable alternative without sympathetic block [19]. To address the gaps in evidence regarding SAPB’s effectiveness in acute regional analgesia following minimally invasive cardiac surgery, this trial evaluates the analgesic effect of SAPB in transapical TAVI using measures such as opioid consumption, analgesic pump pressing times, VAS pain scores at rest and during coughing, and remedial analgesia time. In terms of procedure, ultrasound-guided SAPB requires specialized training and expertise. Ensuring that the researchers performing this procedure are proficient in this technique is crucial for maintaining consistency and accuracy in drug administration. Therefore, establishing ongoing training and certification processes is essential to address this issue. In our current recruitment data, no complications such as bleeding or hematoma have been observed, indicating that SAPB can be safely used in TAVI patients undergoing intraoperative heparinization.

Dexamethasone, a long-acting synthetic glucocorticoid, is utilized as an adjuvant to LAs to extend their duration [20, 21] and mitigate postoperative nausea, vomiting, and chills. In our study, we aim to enhance the duration of analgesia and achieve a more pronounced and sustained effect by combining dexamethasone with ropivacaine. However, it is necessary to monitor the potential side effects of dexamethasone, such as hyperglycemia, especially in this vulnerable patient population. Therefore, a detailed monitoring plan should be established to effectively track and manage these potential adverse effects through blood gas analysis or blood glucose measurement.

Postoperative recovery quality, a complex and dynamic process, is a critical patient-centered outcome, which can be evaluated from the aspects of physiology, psychology, and social adaptation. QOR-40 scale is the best tool to evaluate the quality of early postoperative recovery after clinical intervention based on clinical and research verification. It includes five dimensions: physical comfort, pain, emotional state, physical independence, and psychological support. With good validity, reliability, responsiveness, and clinical acceptability, QOR-40 is widely used in various perioperative environments [13, 22]. Through this comprehensive assessment, our study aims to provide valuable prospective evidence on regional analgesia efficacy in this population. During the recruitment process, we found that ensuring consistent and accurate patient responses can be challenging, especially considering the elderly population involved. To assist patients in completing the QOR-40, our researchers need to use more simplified language to help elderly patients understand, thereby improving data quality.

We acknowledge some limitations in our study. Firstly, our primary outcome is assessed at 24 and 48 h postoperatively, providing insights into early recovery but lacking long-term follow-up to evaluate sustained intervention impact and potential complications. In addition, while we explore the effectiveness of SAPB, uncertainty persists regarding the optimal concentration and dosage of LAs and adjuvants in the block. Future high-quality research is warranted to explore this aspect further, elucidating the most effective drug combinations and furnishing instructive insights for clinical practice.

## Trial status

At the time of manuscript submission, we are conducting this investigation and expect to complete it by January 2025. This is protocol version 1.0, completed on February 24, 2023. Trial recruitment was initiated on February 28, 2023.

## Data Availability

The datasets obtained in the study are available upon reasonable request to the primary researcher.

## Abbreviations

BIS: Bispectral index
CRFs: Case report forms
DMC: Data monitoring committee
DRG: Diagnosis related groups
ERAS: Enhanced recovery after surgery
LAs: Local anesthetics
QoR-40: Quality of Recovery-40 scale
PCA: Patient-controlled analgesia
PCIA: Patient-controlled intravenous analgesia
PLA: Chinese People’s Liberation Army
SAPB: Serratus anterior plane block
TAVI: Transcatheter aortic valve implantation
VAS: Visual analog score

## References

[1] Cribier A, Eltchaninoff H, Bash A, Borenstein N, Tron C, Bauer F, Derumeaux G, Anselme F, Laborde F, Leon MB (2002). Percutaneous transcatheter implantation of an aortic valve prosthesis for calcific aortic stenosis: first human case description. Circulation.

[2] Vahanian A, Alfieri OR, Al-Attar N, Antunes MJ, Bax J, Cormier B, Cribier A, De Jaegere P, Fournial G, Kappetein AP (2008). Transcatheter valve implantation for patients with aortic stenosis: a position statement from the European Association of Cardio-Thoracic Surgery (EACTS) and the European Society of Cardiology (ESC), in collaboration with the European Association of Percutaneous Cardiovascular Interventions (EAPCI). Eur J Cardiothorac Surg.

[3] Walther T, Falk V, Borger MA, Kempfert J, Ender J, Linke A, Schuler G, Mohr FW (2009). Transapical aortic valve implantation in patients requiring redo surgery. Eur J Cardiothorac Surg.

[4] Blanco R, Parras T, McDonnell JG, Prats-Galino A (2013). Serratus plane block: a novel ultrasound-guided thoracic wall nerve block. Anaesthesia.

[5] Mayes J, Davison E, Panahi P, Patten D, Eljelani F, Womack J, Varma M (2016). An anatomical evaluation of the serratus anterior plane block. Anaesthesia.

[6] Perez Herrero MA, Lopez Alvarez S, Fadrique Fuentes A, Manzano Lorefice F, Bartolome Bartolome C (2016). Gonzalez de Zarate J: Quality of postoperative recovery after breast surgery. General anaesthesia combined with paravertebral versus serratus-intercostal block. Rev Esp Anestesiol Reanim.

[7] Kim DH, Oh YJ, Lee JG, Ha D, Chang YJ, Kwak HJ (2018). Efficacy of ultrasound-guided serratus plane block on postoperative quality of recovery and analgesia after video-assisted thoracic surgery: a randomized, triple-blind, placebo-controlled study. Anesth Analg.

[8] Berthoud V, Ellouze O, Bievre T, Konstantinou M, Jazayeri S, Bouchot O, Girard C, Bouhemad B (2018). Serratus anterior plane block for apical TAVR in an awake patient. J Cardiothorac Vasc Anesth.

[9] Peng L, Ding M, Wei W (2023). Ultrasound-guided serratus anterior plane block for transapical transcatheter aortic valve implantation. J Cardiothorac Surg.

[10] Berthoud V, Ellouze O, Nguyen M, Konstantinou M, Aho S, Malapert G, Girard C, Guinot PG, Bouchot O, Bouhemad B (2018). Serratus anterior plane block for minimal invasive heart surgery. BMC Anesthesiol.

[11] Gornall BF, Myles PS, Smith CL, Burke JA, Leslie K, Pereira MJ, Bost JE, Kluivers KB, Nilsson UG, Tanaka Y (2013). Measurement of quality of recovery using the QoR-40: a quantitative systematic review. Br J Anaesth.

[12] Guimaraes-Pereira L, Costa M, Sousa G, Abelha F (2016). Quality of recovery after anaesthesia measured with QoR-40: a prospective observational study. Rev Bras Anestesiol.

[13] Myles PS, Weitkamp B, Jones K, Melick J, Hensen S (2000). Validity and reliability of a postoperative quality of recovery score: the QoR-40. Br J Anaesth.

[14] Dai L, Ling X, Qian Y (2022). Effect of ultrasound-guided transversus abdominis plane block combined with patient-controlled intravenous analgesia on postoperative analgesia after laparoscopic cholecystectomy: a double-blind, randomized controlled trial. J Gastrointest Surg.

[15] Wehrfritz A, Ihmsen H, Fuchte T, Kim M, Kremer S, Weiss A, Schuttler J, Jeleazcov C (2020). Postoperative pain therapy with hydromorphone; comparison of patient-controlled analgesia with target-controlled infusion and standard patient-controlled analgesia: a randomised controlled trial. Eur J Anaesthesiol.

[16] Kukreja P, Uppal V, Kofskey AM, Feinstein J, Northern T, Davis C, Morgan CJ, Kalagara H (2023). Quality of recovery after pericapsular nerve group (PENG) block for primary total hip arthroplasty under spinal anaesthesia: a randomised controlled observer-blinded trial. Br J Anaesth.

[17] Russo M, Corcione N, Cammardella AG, Ranocchi F, Lio A, Saitto G, Nicolo F, Pergolini A, Polizzi V, Ferraro P (2023). Transcatheter aortic valve implantation in patients with age </=70 years: experience from two leading structural heart disease centers. Minerva Cardiol Angiol.

[18] Ancona MB, Toscano E, Moroni F, Ferri LA, Russo F, Bellini B, Sorropago A, Mula C, Festorazzi C, Gamardella M (2021). Patients younger than 70 undergoing transcatheter aortic valve implantation: procedural outcomes and mid-term survival. Int J Cardiol Heart Vasc.

[19] Yu S, Valencia MB, Roques V, Aljure OD (2019). Regional analgesia for minimally invasive cardiac surgery. J Card Surg.

[20] Krom RJ, Welsby IJ, Fuller M, Barbas AS, Gao Q, Anwar IJ, Dunkman WJ (2023). Incidence of postreperfusion hyperfibrinolysis in liver transplantation by donor type and observed treatment strategies. Anesth Analg.

[21] Wu CL, Cho B, Gabriel R, Hurley R, Liu J, Mariano ER, Mathur V, Memtsoudis SG, Grant MC. Addition of dexamethasone to prolong peripheral nerve blocks: a ChatGPT-created narrative review. Reg Anesth Pain Med 2023:rapm-2023-104646. 10.1136/rapm-2023-104646 37295794

[22] Guimaraes-Pereira L, Costa M, Sousa G, Abelha F (2016). Quality of recovery after anaesthesia measured with QoR-40: a prospective observational study. Braz J Anesthesiol.

